# ART to prevent vertical transmission in Latin America: where are we in the “Treat‐All” era?

**DOI:** 10.1002/jia2.26411

**Published:** 2025-04-28

**Authors:** Gabriel Castillo‐Rozas, Fernanda F. Fonseca, Jessica Castilho, Peter F. Rebeiro, Daisy M. Machado, Marco Tulio Luque, Emilia M. Jalil, Fernando Mejia, Ahra Kim, Bryan E. Shepherd, Claudia P. Cortes

**Affiliations:** ^1^ Faculty of Medicine Universidad de Chile Santiago Chile; ^2^ Hospital Clinico San Borja Arriaran & Fundación Arriaran Santiago Chile; ^3^ AIDS HealthCare Foundation, Global Program São Paulo Brazil; ^4^ Division of Infectious Diseases Department of Medicine Vanderbilt University Medical Center Nashville Tennessee USA; ^5^ UNIFESP, Escola Paulista de Medicina, Disciplina de Infectologia Pediátrica São Paulo Brazil; ^6^ Hospital Escuela Universitario Tegucigalpa Honduras; ^7^ Instituto Nacional de Infectologia ‐ Evandro Chagas, Fiocruz Rio de Janeiro Brazil; ^8^ Instituto de Medicina Tropical Alexander von Humboldt Universidad Peruana Cayetano Heredia Lima Perú; ^9^ Department of Biostatistics Vanderbilt University Medical Center Nashville Tennessee USA

**Keywords:** HIV epidemiology, Latin America, pregnancy retention, vertical transmission, women, newborn

## Abstract

**Introduction:**

Antiretroviral therapy (ART) during pregnancy and at delivery has nearly eliminated vertical transmission (VT) in some settings but previously reported VT prevalence has been as high as 15% in Latin America and the Caribbean (LAC). We evaluated VT in the Caribbean, Central and South America network for HIV epidemiology to further study the benefit of ART on VT in our region.

**Methods:**

We retrospectively collected data on cis‐gender women ≥15 years of age enrolled in HIV clinics in Brazil, Chile, Honduras and Peru from 2003 to 2018 with ≥1 pregnancy resulting in a live birth after clinic entry to examine the association of ART use at the time of delivery and VT. We used propensity‐score‐matched logistic regression to examine the odds of VT by ART use. Matching weights incorporated site, HIV RNA, CD4 cell count, maternal age, year and HIV diagnosis before or during pregnancy. We also examined the proportion of women who received ART during pregnancy before and after the treat‐all era, as defined within each country.

**Results:**

A total of 623 pregnant women with HIV contributed 727 live births. Of all births, 613 (84.3%) infants had known HIV status and there were 22 (3.6%) VT events. Four of the 22 (18%) were born to women on ART at delivery, compared to 403 of 591 (68%) infants negative for HIV. In the propensity‐score‐matched model, ART use at delivery was associated with 85% decreased odds of VT (odds ratio = 0.15, 95% confidence interval 0.04−0.58). In the pre‐treat‐all era, 37% (181/485) of women received ART within 30 days of pregnancy diagnosis, compared to 59% (75/128) during the treat‐all era (*p*<0.001). In the pre‐treat‐all era, 4.3% (21/485) of infants were born HIV positive, compared to 0.8% (1/128) in the treat‐all era (*p* = 0.055).

**Conclusions:**

We found a low prevalence of VT in our cohort, especially in the treat‐all era. ART use at delivery was strongly associated with a lower odd of VT. Despite improvements, access to ART during pregnancy remained far from universal. Therefore, new strategies to ensure its effective implementation in LAC are still warranted.

## 1 INTRODUCTION

The prevention of HIV vertical transmission (HIV‐VT) has been a major achievement in the public health response to the HIV epidemics. Pioneering early studies demonstrated that the administration of zidovudine at the time of delivery resulted in a 67.5% reduction of VT (ACTG 076 trial [1]). Along with this efficacious intervention, several strategies can and should be implemented to prevent HIV acquisition in infants exposed to the virus, such as stop breastfeeding (when it is feasible), assure universal access and adherence of women to antiretroviral therapy (ART) and to stratify the risk of infants of acquiring HIV according to their individual and community health determinants to design the best‐tailored postnatal prophylaxis, as proposed by Penazzato et al. last year on this Journal [2].

In fact, and due to the use of ART during pregnancy, the annual incidence of VT decreased from 42% in the 1990s [3] to approximately 10% globally in 2023 [4, 5]. This global success has been possible due to the widening of ART access: its coverage among pregnant women with HIV has significantly increased in the last decade. While only 48% of pregnant and breastfeeding women were receiving ART in 2010, approximately 82% of pregnant or breastfeeding women living with HIV are now receiving ART, according to the 2023 UNAIDS report [5]. This progress has allowed to avert 3.4 million HIV acquisitions in children since 2020 [6] and to reduce in incidence of VT almost to zero in some countries [6].

However, the epidemiological situation is not homogeneous around the globe. While only 19 countries have officially stopped HIV‐VT, most of the world still strives to prevent these events. Particularly, in Latin America and the Caribbean (LAC), we face different challenges among countries. Although 11 of the 19 countries that have stopped VT are in the Americas, our region reaches a worrisome VT rate of 15% according to the 2024 UNAIDS report [5]. Furthermore, the uptake of ART and successful prevention of VT has not been consistent worldwide. ART coverage for pregnant women and HIV screening in exposed infants requires attention and improvement in LAC, in particular. As recently as 2024, only 63% of pregnant women in Latin America were receiving ART [5] and only 46% of exposed infants were screened for HIV by 8 weeks of age [7]. It is worth noting, however, that this data considers the entire region, and we have not yet analysed these parameters among the sites of our consortium across the years and considering the changes in policies for ART initiation criteria.

The need to assess the epidemiology of VT in LAC led us to study pregnancy outcomes in the Caribbean, Central and South America network for HIV epidemiology (CCASAnet). CCASAnet is the largest and most comprehensive data source of people living with HIV who receive medical care in the region. In this study, we examined the effect of receiving ART during pregnancy and at delivery on VT events, among pregnant women with HIV in four HIV clinic centres in LAC from 2003 to 2018.

## 2 METHODS

### 2.1 Study population

In this retrospective cohort study, we included all cis‐gender women with HIV aged ≥15 years and enrolled in HIV care at CCASAnet sites between 2003 and 2018 who had available data and at least one pregnancy that occurred after clinic entry and that resulted in a live birth. Participants were eligible to contribute to multiple pregnancies during the study period. CCASAnet sites participating in this study included: *Instituto Nacional de Infectologia Evandro Chagas* (Brazil), *Universidade Federal de São Paulo* (Brazil), *Fundación Arriarán* (Chile), *Hospital Escuela & Instituto Hondureño de Seguridad Social* (Honduras) and *Instituto de Medicina Tropical Alexander von Humboldt* (Peru). Pregnancy events occurring among eligible women were identified by a detailed medical chart review. Research staff at participating sites abstracted additional pregnancy‐related data elements from medical records at HIV and prenatal clinics using standardized data collection forms. Pregnancy outcome data, including HIV testing results from live births, were obtained from thorough medical record reviews at each site, as well as self‐report from mothers in case the HIV testing was done in a different clinic. If there was more than one pregnancy, the mother's demographic data was recorded only for the first event. To obtain information on the national guidelines for preventing VT during pregnancy events during the follow‐up period, we summarized recommendations according to the documents provided by investigators from each site included in the cohort. The ethical approval and the informed consent waiver for the use of these data were granted at each of the CCASAnet sites and at Vanderbilt University Medical Center (IRB #060284).

### 2.2 Outcome

The primary outcome was the HIV status of newborn infants (HIV positive, HIV negative, or missing data) as measured by HIV RNA polymerase chain reaction (PCR) and with verification mechanisms depending on the official regulations of each country or reported by the caregiver in case of testing performed outside the study site. The absence of a positive or confirmatory test in each centre was considered as a negative HIV status. Finally, infants with unknown HIV status were excluded from the analyses.

### 2.3 Exposure, risk factors and potential confounders

Our primary exposure of interest was the administration of combination ART during pregnancy and at the time of delivery. Additional risk factors and potential confounders for VT included type of ART regimen (defined by core agents as protease inhibitor [PI]‐based, non‐nucleoside reverse transcriptase inhibitor [NNRTI]‐based or integrase inhibitor [II]‐based), HIV‐1 RNA viral load (VL, log_10_ copies/ml) closest to delivery (± 30 days); CD4 cell count (cells/µl) closest to delivery (± 30 days); and calendar year of delivery. Guidelines for preventing VT in pregnant women varied over the course of the study and across countries. From our observational data, it was not possible to classify women or their clinical providers as following or not following specific treatment guidelines during pregnancy. Instead, we evaluated whether women received ART during pregnancy or at the time of delivery based on when treat‐all guidelines were adopted in each country (1 January 2013, for Honduras; 1 January 2015, for Brazil; and 1 September 2015, for Chile and Peru).

### 2.4 Statistical analysis

We described the characteristics of all pregnant women included in the study using frequencies (proportions) for categorical variables and medians (interquartile ranges [IQRs]) for continuous measures. To assess the crude associations between the mothers’ demographic and clinical characteristics (exposure variables) and newborn HIV status (outcome variable), we conducted simple logistic regression with the key covariates described above. Because the number of infants born with HIV was small, we fit a propensity score‐adjusted model to examine our primary question regarding the role of ART use during pregnancy and at delivery and the risk of VT. The logistic regression model to construct propensity scores used women's ART status at the time of delivery as the outcome variable. As predictors, we included site, maternal HIV VL at delivery, square‐root‐transformed CD4 cell count at delivery, year of delivery, maternal age at delivery and whether a woman was diagnosed with HIV before or after pregnancy status was determined. To account for missing data, we conducted multiple imputation using chained equations with 20 replications prior to creating propensity scores. We obtained matching weights for each imputed dataset, and we conducted the main analysis predicting newborn HIV status on each imputed dataset. Each participant was eligible to contribute more than one pregnancy to the analysis; robust standard errors were computed for each imputed dataset. Coefficients and standard errors were combined across imputed datasets using Rubin's rules, and confidence intervals were then calculated.

Study data were collected and managed using REDCap version 8.9.2 hosted at Vanderbilt University Medical Center [8, 9]. REDCap (Research Electronic Data Capture) is a secure, web‐based software platform designed to support data capture for research studies, providing (1) an intuitive interface for validated data capture; (2) audit trails for tracking data manipulation and export procedures; (3) automated export procedures for seamless data downloads to common statistical packages; and (4) procedures for data integration and interoperability with external sources. Analyses were performed using R statistical software version 4.2.1.

## 3 RESULTS

A total of 623 pregnant women with HIV who had 727 live births were included in the study population: 211 (33.9%) women and 285 (39.2%) infants from Brazil, 91 (14.6%) women and 98 (13.5%) infants from Chile, 90 (14.4%) women and 93 (12.8%) infants from Honduras, and 231 (37.1%) women and 251 (34.5%) infants from Peru. Relevant characteristics of participants are described in Table 1. The median year of HIV diagnosis was 2009 and the median year of first pregnancy after clinic enrolment was 2012. The median age at first pregnancy was 27.3 years. Most women (68.6%) were diagnosed with HIV before pregnancy and 27.5% during pregnancy. With regard to ART use, 40.9% of women were receiving ART prior to pregnancy diagnosis, 38% initiated ART during pregnancy and 15.4% never started ART. Most deliveries were caesarean (C)‐sections (84.2%).

**Table 1:** Characteristics of the pregnant women from the cohort (2003−2018)

	*N*	All (*n* = 623)
**Country**	623	
CCASAnet‐Brazil		211/623 (33.9)
CCASAnet‐Chile		91/623 (14.6)
CCASAnet‐Honduras		90/623 (14.4)
CCASAnet‐Peru		231/623 (37.1)
**Year of HIV diagnosis**	620	
Median (interquartile range)		2009 (2006−2013)
<2003		37/620 (6.0)
2003–2010		338/620 (54.5)
2011–2015		174/620 (28.1)
2016–2018		71/620 (11.5)
**Earliest ART year**	590	
Median (interquartile range)		2011 (2007−2014)
<2003		7/590 (1.2)
2003–2010		281/590 (47.6)
2011–2015		225/590 (38.1)
2016–2018		77/590 (13.1)
**Year of first pregnancy**	623	
Median (interquartile range)		2012 (2008−2015)
<2003		2/623 (0.3)
2003–2010		270/623 (43.3)
2011–2015		248/623 (39.8)
2016–2018		103/623 (16.5)
**Years from HIV diagnosis to first pregnancy diagnosis**		
Median (interquartile range)		0 (0−3)
**Years from ART initiation to first delivery**		
Median (interquartile range)		0 (0−2)
**Number of pregnancies**	623	
Median (interquartile range)		1 (1−1)
**Age at first pregnancy**	623	
Median (interquartile range)		27.3 (23.1−32.3)
**CD4 at pregnancy diagnosis**	273	
Median (interquartile range)		371 (209−560)
**CD4 at delivery**	219	
Median (interquartile range)		483 (311−740)
**Viral load (Log10 RNA) at pregnancy diagnosis**	247	
Median (interquartile range)		4 (2−4)
**Viral load (Log10 RNA) at delivery**	360	
Median (interquartile range)		2 (2−2)
**Detectable viral load at pregnancy: Detectable**	247	178/247 (72.1)
**Detectable viral load at delivery: Detectable**	360	47/360 (13.1)

*Note*: *N* is the number of non‐missing value. For women with multiple pregnancies, only the first observation in the data was included.

Of the 727 infants included in the database, 114 (16%) had an unknown HIV status. Although all sites performed HIV RNA PCR to diagnose or rule out HIV infection in infants, only 134 (18%) test results were recorded in the database. The remaining known infants’ HIV statuses (*n* = 479, 66%) were reported by caregivers, and presumably corresponded to HIV RNA PCR tests.

Table 2 summarizes the characteristics of the 613 infants with known HIV status (84% of total live births). The median year of delivery for infants with HIV was 2009 compared to 2012 for infants without HIV. Women were prescribed ART at the time of delivery for 4 of the 22 (18%) infants diagnosed with HIV, compared with 403 of 591 (68%) infants who were negative for HIV. Maternal HIV viral load at the time of delivery was missing for a substantial percentage of births: 60% (15/22) of infants diagnosed with HIV and 42% (249/591) of infants who were HIV negative. Among women with available results near the time of delivery, HIV viral load was undetectable for 71% (5/7) of mothers with infants diagnosed with HIV compared to 87% (297/342) of mothers of infants without HIV (*p* = 0.66). In both cases of infants diagnosed with HIV to mothers with detectable viral loads, the women were diagnosed with HIV at or after the pregnancy diagnosis and both women started ART after delivery, which occurred in 2005 and 2012. Of the five mothers with undetectable viral load whose children became HIV positive, two were diagnosed with HIV <50 days before delivery. One of these mothers started ART immediately when diagnosed with HIV, but was already >200 days into their pregnancy; the other, whose baby was delivered in 2006, did not start ART until after delivery. The other three women with undetectable viral load at delivery but with HIV‐positive infants all started ART at or prior to pregnancy diagnosis.

**Table 2:** Characteristics of the pregnant women included in analysis stratified by infant HIV status during the observation period (2003−2018)

	*N*	Negative (*n* = 591)	Positive (*n* = 22)	Overall (*n* = 613)	*p* value
**Country**	613				*p =* 0.22^b^
Brazil		215/591 (36.4)	9/22 (40.9)	224/613 (36.5)	
Chile		86/591 (14.6)	6/22 (27.3)	92/613 (15.0)	
Honduras		75/591 (12.7)	3/22 (13.6)	78/613 (12.7)	
Peru		215/591 (36.4)	4/22 (18.2)	219/613 (35.7)	
**On ART at time of delivery**	613				*p<*0.01
No		188/591 (31.8)	18/22 (81.8)	206/613 (33.6)	
Yes		403/591 (68.2)	4/22 (18.2)	407/613 (66.4)	
**CD4 T cell count at delivery**	210				*p =* 0.65^a,b^
Median (interquartile range)		526 (333−737)	503 (450−810)	526 (336−743)	
**Detectable viral load at delivery**	349				*p =* 0.24^a^
Undetectable		297/342 (86.8)	5/7 (71.4)	302/349 (86.5)	
Detectable		45/342 (13.2)	2/7 (28.6)	47/349 (13.5)	
**Regimen class closest to delivery**	407				*p =* 0.01a
PI‐based		258/403 (64.0)	1/4 (25.0)	259/407 (63.6)	
IINSTI/Other		30/403 (7.4)	2/4 (50.0)	32/407 (7.9)	
NNRTI‐based		115/403 (28.5)	1/4 (25.0)	116/407 (28.5)	
**Year of delivery**	613				*p<*0.01^b^
Median (interquartile range)		2012 (2008−2015)	2009 (2006−2011)	2012 (2008−2015)	

*Note*: *N* is the number of non‐missing value.

^a^ Pearson’s chi‐squared test.

^b^ Wilcoxon.

**Figure 1:**
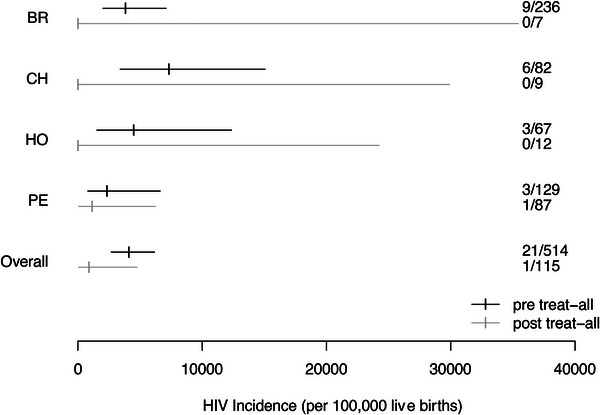
HIV‐VT incidence estimates per site and according to the time (“pre‐treat‐all era” vs. “treat‐all era”). BR, Brazil; CH, Chile; HO, Honduras; PE, Peru.

Among the 613 women whose infants had known HIV status, 37% (181/485) were on ART within 30 days of their pregnancy diagnosis during the pre‐treat‐all era compared to 59% (75/128) after the treatment guidelines changed (*p*<0.001). In the pre‐treat‐all era, 4.3% (21/485) of infants were diagnosed with HIV, compared to 0.8% (1/128) in the treat‐all era (*p* = 0.055). The single infant diagnosed with HIV in the treat‐all era was born to a mother who was not on ART within 30 days of her pregnancy diagnosis but who had started ART prior to delivery. Table 3 shows the number of infants diagnosed with HIV according to if the VT events occurred prior to or during the “treat‐all era.” Moreover, Figure 1 illustrates the estimates of HIV‐VT incidence according to our results.

**Table 3:** Numbers of infants diagnosed with HIV among live births of women living with HIV according to the site and to the “treat‐all era” (2003−2018)

Country	Pre‐treat‐all era	Treat‐all era
Brazil	9/236 (3814; 2019–7088)	0/7 (0; 0–35,433)
Chile	6/82 (7317; 3396–15,058)	0/9 (0; 0–29,915)
Honduras	3/67 (4478; 1534–12,358)	0/12 (0; 0–24,249)
Peru	3/129 (2326; 794–6614)	1/87 (1149; 59–6227)
**Overall**	**21/514 (4086; 2688–6165)**	**1/115 (870; 45–4762**)

*Note*: The numbers are expressed in terms of the pregnancies produced per site. In parenthesis, they are expressed as number per 100,000 live births and with their 95% confidence intervals.

The values in bold were placed in this format to emphasize that it is the result of the total set.

In the propensity score model, if the mother was receiving ART at the time of delivery, the weighted odds of the infant being HIV positive decreased by 85% (weighted odds ratio [OR] = 0.15, 95% confidence interval [CI] 0.04−0.58, *p* = 0.006). This association was similar when we only assessed the first pregnancy for each mother (*n* = 623, OR = 0.15, 95% CI 0.04−0.58, *p* = 0.006).

To better understand temporal trends in outcomes during the observation period, we constructed a comparative table including national treatment guidelines for the countries included in this cohort (Table 4) [10−19]. We observed differences in the timing of treat‐all implementation as well as differences in first‐line ART regimens recommended for pregnant women. The diagnostic strategy for newborns was not specified in all guidelines and there was a consensus for avoiding breastfeeding.

**Table 4:** Local guidelines for HIV‐vertical transmission prevention published during the observation period in the included countries (2003−2018)

	Honduras (2008) [9]	Honduras (2013) [10]	Brazil (2006) [11]	Brazil (2010) [12]	Brazil (2015) [13]	Peru (2005) [14]	Peru (2008) [15]	Peru (2015) [16]	Chile (2005) [17]	Chile (2012) [18]
**ART initiation in every pregnant woman?** ^a^	No	Yes	No	No	Yes	No	No	No	No	No
**Scheme of choice for pregnant women**	AZT/3TC + LPV/r as prophylaxis^b^	TDF/FTC + EFV	Not specified	AZT/3TC + LPV/r	TDF/3TC + EFV	AZT as prophylaxis^b^	AZT+3TC+LPV/r	TDF/3TC/EFV	AZT/3TC + ritonavir‐boosted PI	AZT/3TC + LPV/r
**Vaginal delivery possible in women with low viraemia?**	No	No	Yes	Yes	Yes	Yes	Yes	No	Yes	Yes
**Parenteral AZT protocol specified?**	No	Yes	Yes	Yes	Yes	No	No	Yes	Yes	Yes
**Breastfeeding possible?**	Yes	No	No	No	No	No	No	No	No	No
**Diagnostic workup specified for infants?**	No	Yes	Yes	No	No	Yes	No	No	Yes	Yes
**HIV PCR scheme in infants**	−	72 hours, 1 month, 6 months	1 and 4 months	−	−	6 months	−	−	48 hours, 15–30 days, 3 months	48 hours, 15–30 days, 3 months
**HIV serology included?**	−	No	At 12 months if both PCRs are (−)	−	−	At 18 months	−	−	No	No

Abbreviations: ART, antiretroviral therapy; AZT, zidovudine; EFV, efavirenz; FTC, emtricitabine; HIV‐VT, HIV vertical transmission; LPV/r, ritonavir‐boosted lopinavir; PCR, polymerase chain reaction; PI, protease inhibitor; TDF, tenofovir disoproxil fumarate; 3TC, lamivudine.

^a^ For women diagnosed with HIV during pregnancy.

^b^ For women not meeting criteria for ART initiation, aiming to reduce VT.

## 4 DISCUSSION

In this retrospective cohort study of pregnant women receiving HIV care in four centres of LAC from 2003 to 2018, we observed a low prevalence of VT, with a decrease from the pre‐treat‐all to the treat‐all era. The prevalence of VT was 3.6% in our analysis, with the highest estimate in Chile (6.5%), followed by Brazil (4.0%), Honduras (3.8%) and Peru (1.8%). There was a substantial decrease in VT in the treat‐all era (0.8%) compared to the pre‐treat‐all era (4.3%). We identified a strong protective effect of ART on VT risk. To our knowledge, this is the first data on VT after the adoption of treat‐all recommendations in LAC.

Our findings are consistent with previous studies that established combination ART as the primary mode of prevention for VT [5, 20, 21]. Since the groundbreaking ACTG 076 trial, which demonstrated a nearly 70% reduction of VT by administering zidovudine during pregnancy and labour combined with a short course of ARV to infants after birth [1], ART use has been playing a critical role in reducing VT. In fact, in women receiving ART and with sustained undetectable VL during pregnancy and at delivery, the administration of intravenous zidovudine did not add further benefit in preventing VT [22]. This finding highlights the relevance of ART initiation in pregnant women as soon as possible and to maintain adherence to ART.

In our cohort, women whose infants were diagnosed with HIV had lower rates of ART use at delivery, lower rates of HIV undetectability at delivery and higher rates of missing HIV viral load data at delivery. However, even among infants who were HIV negative, many women in our cohort had worrisome HIV indicators at delivery: 10% of them were not virologically suppressed and 42% were missing HIV RNA measurements. Despite not being among the most affected by HIV in LAC, women may have worse outcomes than men, with lower ART uptake [23, 24]. These facts should serve as a warning for regional governments and healthcare providers that ART must be started as soon as possible for all women regardless of pregnancy status and reproductive planning, and its adherence carefully monitored in every pregnant woman to prevent VT events, and, therefore, to stop VT in our region.

Our study's consideration of the treat‐all era also highlighted the importance of implementing universal ART initiation in preventing VT. Our results show a worrisome ART coverage in pregnant women during the observation period; and although it improved across the years, it still lags behind the coverage in pregnant women reported by UNAIDS in the last report [25]. Of the 22 infants diagnosed with HIV, only one occurred after the treat‐all era began. After all, a cornerstone of HIV prevention and optimal prenatal care is timely HIV screening, diagnosis, and, in the treat‐all era, ART initiation regardless of CD4 count, clinical stage and pregnancy status [26]. This strategy enables to start ART as soon as possible to reach virologic suppression prior to delivery.

In this study, we identified that clinical guidelines for preventing VT varied significantly across LAC over time and often lagged behind international guidelines. We recommend governments regularly update their national guidelines according to the most recent scientific evidence to address VT as an urgent public health concern [27]. Additionally, local Ministries of Health should strictly monitor clinical adherence to local guidelines, promoting the development of improvement strategies when necessary.

Furthermore, we observed a high frequency of C‐section despite the low proportion of women with detectable VL at delivery. Considering the higher risk of complications with C‐section in comparison to vaginal delivery, and the fact that vaginal delivery has been shown to be safe for women with low VL [28], intensive coordination and education with obstetric teams is warranted to improve adherence to international guidelines and recommendations.

Some limitations in our study must be acknowledged. The low prevalence of VT in our study compared to previous data in the region may be a result of including referral and specialized centres in our cohort and including only women with HIV who had been successfully linked to care. Therefore, these results may not be generalizable to all LAC settings and populations. Second, we were unable to assess adherence to national VT prevention guidelines due to incomplete data captured in medical records. To account for this limitation, we focused on the treat‐all era as a landmark shift in HIV treatment more generally. Additionally, we observed high rates of missingness for HIV VL at delivery and infant HIV status, which might have biased our results. Lastly, we acknowledge the possibility of information bias at different levels: first, as the sites could have failed to record all the pregnant women considering the way the pregnancy data were collected; second, as most infants’ HIV statuses were classified according to caregiver report; and, finally, as some misinformation might be secondary to loss‐to‐follow‐up or transfer of pregnant or postpartum women. These possible situations could have influenced the incidence of VT measured in this cohort and the magnitude and direction of detected associations; however, the false negative rate is likely negligible considering the regular monitoring of positive PCR results conducted in the respective centres.

Those limitations concerning the loss to follow‐up of pregnant and postpartum women should serve as a warning for the governments and decision‐makers. Some of these women were transferred to other clinics according to their own decisions, but also some of them might have stopped adhering to ART medication for multifactorial reasons, thereby affecting the woman and her newborn infant's health. Regardless of the reason, the institutions in charge should incorporate robust social services inside HIV clinic centres that are able to monitor linkage to care and to track down women who leave care in a timely fashion to avoid viral rebound in the women and the VT to their newborns.

Finally, data collection stopped during the COVID‐19 pandemic due to a shortage of research resources, thus we are not able to illustrate a more updated situation of our centres. Therefore, future research is warranted to address this gap. Nevertheless, and even when we have observed a trend towards the normalization of our healthcare services during the “post‐pandemic period,” we still face challenges as those previously discussed in this section and our centres (and countries) are still working for a fully normalization of the pregnancy and postpartum‐associated healthcare services to prevent HIV VT.

## 5 CONCLUSIONS

In conclusion, our study reports a lower HIV VT rate than that reported by UNAIDS for our region and shows the real‐world effectiveness of ART use during pregnancy to prevent VT in specialized centres for HIV care in Latin America. However, these data are not representative of the differences in our region's healthcare challenges. Despite this limitation of external validity, the ART coverage among pregnant women in LAC is still lagging behind even in the treat‐all era. Therefore, it is of utmost importance for governments to ensure broad and effective access to ART for pregnant women in LAC and to ensure the proper linkage to care to prevent any HIV VT event, as proposed by the 2030 Sustainable Development Goals [29]. Further research is warranted to study and comprehend the different factors involved in the low coverage of ART during pregnancy in order to design locally adjusted public health policies.

## COMPETING INTERESTS

All authors declare no conflicts beyond the funding listed above.

## AUTHORS’ CONTRIBUTIONS

CPC designed the research study. AK and BES performed the statistical analyses. GC‐R and CPC reviewed critically and summarized the local guidelines on VT prevention. GC‐R, FFF, JC, PFR, DMM and CPC wrote the manuscript. All the authors reviewed the manuscript critically and approved its final version.

## FUNDING

This work was supported by the NIH‐funded Caribbean, Central and South America network for HIV epidemiology (CCASAnet), a member cohort of the International epidemiology Databases to Evaluate AIDS (leDEA) (U01AI069923). This award is funded by the following institutes: the National Institute of Allergy and Infectious Diseases (NIAID), the Eunice Kennedy Shriver National Institute of Child Health and Human Development (NICHD), the National Cancer Institute (NCI), the National Institute of Mental Health (NIMH), the National Institute on Drug Abuse (NIDA), the National Heart, Lung, and Blood Institute (NHLBI), the National Institute on Alcohol Abuse and Alcoholism (NIAAA), the National Institute of Diabetes and Digestive and Kidney Diseases (NIDDK), the Fogarty International Center (FIC), the National Library of Medicine (NLM) and NCATS/NIH (UL1TR000445).

## DISCLAIMER

The content is solely the responsibility of the authors and does not necessarily represent the official views of the National Institutes of Health.

## Data Availability

The data that support the findings of this study are available on request from the corresponding author. The data are not publicly available due to privacy or ethical restrictions.
